# The Bible

**Published:** 1870-04

**Authors:** 


### THE BIBLE

Is of divine origin. Its Author is God. He did not write it himself. He dictated it. He influenced good men to write it. He caused a desire of writing to come over them, and they wrote — as we are impelled to write letters to kindred and dear friends far away, by some vivid remembrance of them. Under such circumstances, we may write what is not true; but holy men of old wrote as they were moved by the Almighty Mind, who could not have moved them to write one syllable which was not strictly true in all its bearings.

God is the author of one book — The Bible: man, of many. His book needed no second edition, because it contained all that was necessary to be known on the great subject of which it treated; and all it did communicate was true, without any admixture of error.

Not so with the work of man. No sooner is it written, than he discovers mortifying errors and inaccuracies, and he alters and amends until he dies. As years pass on, and as human knowledge progresses, even our standard scientific works are found to have less and less of truth, more and more of error. Not so with the holy volume. It is the very reverse. Progressive years, and ages, and millennials are evolving new truths, in proportion as it is more closely studied. It takes for granted, speaks of as a familiar thing passing before the mind's eye of writers, thousands of years ago, what is new to us, this very Anno Domini eighteen hundred and seventy. Assertions made in the earlier ages of the world's history as to what had been, was then, and would be, are even now being verified by facts which the current history of events is constantly eliminating.

Among these biblical assertions are: that less remote than the earth's creation was that of living creatures; at a later date, plants; and later still was man, a few thousand years ago only. The laborious researches of geology afford us, at length, and recently, this demonstration.

The surface of the earth, for the depth of eight or ten miles, is ascertained to be composed of rocks. Very low down, these rocks contain the remains of no living thing, whether of animal, plant, or man. At a less depth, various animal relics are found, to the number of thirty thousand different kinds; and this side, there are countless vegetable fossils; but among them no remains, however small, of any human creature, has ever been discovered. But the bones of animals and men are of the same structure, and of the same constituents, and would endure equally long under the same conditions; but as not a single human bone is found among these thirty thousand kinds of fossil remains of animal life, we are perfectly sure that animals lived before man, as is declared in the first chapter of *Genesis*. But it is only within a very few years that the materials for this most impregnable argument have been gathered.

Further, among these relics of breathing life, in the far back ages, when chaos reigned, and impenetrable darkness brooded in silent grandeur over the planet on which we now dwell, there is no remains of vegetation, not a leaf, or twig, or tree, because they could not grow in darkness; hence we find that these remains are at a less depth still: and even among these, there is not one single relic of humanity. Thus it is that the Bible declares, with beautiful and amazing accuracy, that the waters existed in darkness, that light came, then vegetation appeared, in the order of grass, and herb, and tree, precisely as is observed in our own time, in the reclaimed lands at the mouths of great rivers, of which the Mississippi is a familiar illustration to many of our readers.

After reptiles, and fishes, and vegetation, and animals, Man came. Thus it is that his remains are found but a short distance under ground, in the *alluvium*, the "filling up" of the last hundred feet of the earth's surface, which, of necessity, must have been of comparatively recent occurrence.

In this way, the accumulated acquisitions of science, culminating in a point, in the middle of this nineteenth century, demonstrate the truth of a Bible statement, made six thousand years ago, and which very few of us, until within the last twenty-five years, thought possible of proof. That the telegraph and steam-car were foreshadowed in the same far-seeing record, seems very probable. Twenty-five hundred years ago, *Nahum* (ii. 4) wrote of chariots seeming like torches, and that they should run like the lightning, with terrible collisions in the highways; and earlier still, by near a thousand years, *Job* (xxxviii. 35) inquires if the lightnings could be sent to convey intelligence? — three millennials pass away, and *Morse* responds "THEY CAN!"

It must, then, strike the reader with great force, that a book so minutely, so grandly accurate, in the use of scientific truths, of one class, is equally worthy of reliance on all other subjects; and that whatever it announces must be founded on eternal truth, — consequently can be leaned on with most perfect safety; and therefore, as to whatever it may say as to practical life, should be far more eagerly sought for than that fabled *Philosopher's Stone*, which was to turn all it touched into the finest gold. What are the Bible teachings as to human health? He who lives wisely, shall live long;

"*shall come to his grave in a full age, like as a shock of corn cometh in his season.*"

What is it then to live wisely? Let that same conservative volume reply, in its own identical language, when it declares a conceded fact as to the means of attaining physical perfection, and power, and skill. "*Every man that striveth for the mastery* (*in the Olympian Games, in racing, wrestling, etc.*), *is temperate in all things.*" And that we might not be left in doubt as to what the nature of that temperance is, we have a word from the same Greek foundation in another connection: "*Let your moderation be known unto all men;*" both words meaning self-restraint, self command, self-government. We are to look at all things, whether of theory or of practice, in a calm, and quiet, and sober light,— to take a medium course.

How does this wide-reaching principle dash to atoms the multitudinous and baseless fabrics of the times, the thousand and one isms which disturb the Church, and State, and social life, under the deceptive name of "Reform," which, in ninety-nine cases out of a hundred, is the flag of the devil, the rallying-point, in politics, and medicine, and religion, of the educated knave, the pitiful ignoramus, and the heartless hypocrite.

This "Temperance," this Divine "Moderation," is wide-reaching in its application: it extends to all that we eat, and drink, and do. It does not mean that we are to die of thirst, nor to live on thin air, but that we are to partake of all the good things of this life, as rational beings. We are to eat in moderation; drink in moderation; sleep in moderation; exercise in moderation. In these four things consist human health, human happiness, and human success.

One of the broadest foundations of the Bible is laid in the remarkable fact, that, to a very wide extent, in the Jewish Theocracy, *the means of health was made a part of their religion.* We have for more than a quarter of a century wanted to hear some great and competent mind preach a scriptural sermon on that subject, one which should have chapter and verse, and undisputed whole facts to back every enunciation, for that is the preaching of power, the world over, convincing alike the civilized and the savage; it is the terror to evil-doers, and the encouragement, and upholding, and strengthening of them who do well.

Next to temperance, as a means of health, is cleanliness in person, clothing, and habitation. Hence one of the very first religious rites was that of circumcision — the immediate effect of which is the promotion of personal cleanliness; and by its cooling and diminishing the exquisite sensibility of the parts, it largely modifies the vicious tendencies which are the bane of the young; and more than that, does much towards preventing those exhaustions which afflict the unmarried. And, further, we have never known or heard of a case where a Jew ever committed suicide within a day or two after marriage.

The very specific directions in the twelfth chapter of Leviticus as to the purification of the parturient, are as needful this day as to personal cleanliness as in the earliest ages of the world's history. There should be the same sanctity now about such things that there was in Moses's time.

With the same intent, in part — the promotion of personal cleanliness, — were the injunctions against touching the carcasses of carrion beasts and birds, the penalty being that the person should remain *unclean* the remainder of the day; unfit to associate with others.

The appearance of certain reddish or greenish streaks in the walls of a house, especially if inclined to spread, was to be an indication that such a house was unfit for a human habitation; and the command was imperative, that it should be torn down, stones, timbers, everything, and scattered abroad. We know that appearances of this kind in houses, indicate decomposition; and that there must be moisture, dampness, there: and the most uninformed know that a damp house, or a damp locality, is unfavorable to health.

As to the plague of leprosy, it was not only fearful in its ravages in the system, but the restrictions imposed on the unfortunate persons who were its victims, made it, if possible, a still more terrible malady. To be an outcast from society; to come in contact with no living soul; to be shunned instinctively by every one; and, lest persons should approach unawares, to be compelled to give the loud warning to all coming near, " Keep away, I am 'unclean,' unfit to associate with my kind!" A disease of which there was no hope of cure. The skin became dry and scaly; the hair fell out; the eyebrows and eyelashes dropped off; the nails of fingers and feet were eaten away; ugly excrescences and putrid sores deformed the person; and by imperceptible, yet resistless advances, the miserable body, joint by joint, was eaten away, yet not to be arrested by natural death, until the corrupted mass before you had scarcely a relic to indicate that it had ever been human.

It is reasonable to suppose that leprosy now is the same in its general nature as in ancient times; and, taking this for granted, we have only to make use of a common observation as to lepers in Oriental climes, as also those of higher latitudes — that it is owing in large part to *want of personal cleanliness,* and that, terrible as it is, it is the deserved curse of the more than beastly filthiness in which the wretched creatures wallow.

Abstinence from the use of lard, and pork meat, and other gross food, with weekly fastings and personal ablutions, imposed on the Hebrew nation, have largely aided in making them a healthy and prolific people, in every portion of the globe — exempting them, to a great extent, from the plagues and pestilences which have depopulated other nations. Doubtless, it was in anticipation, in part, of their to be scattered condition, that these precepts were made part and parcel of their religion, as a means of preserving them a peculiar people to Himself — a people whose greatest glory is yet to come, and will not tarry; and for the accomplishment of whose preservation, in health and numbers, in spite of exposure to the diseases of every clime, Divinity has ordered the strict observance of the fundamental principles of Hygiene. It was upon cleanliness and temperance that the great Howard relied as protectors against noisome dungeons and the plagues of the Orient. Nor can we as well account for the remarkable fact, that at this hour, the most filthy part of modern

Rome, the Ghetto, with its dilapidated houses, and odorous atmosphere, is made, by law, the Hebrew quarter; and yet to them it is not an unhealthy locality — presenting a striking exemplification of that Divine beneficence, which, while it makes obedience a test of fidelity, causes that obedience to be followed by a direct blessing, the blessing of bodily health. And so might we speak of the numerous purgations, by water and fire, which occupy so large a space in Mosaic history — all designed in their bearings to promote purity of body, purity of clothing, purity of habitation — all leading upwards to a higher and holier end, purity of heart and soul, for now and for aye.

Physicians of all schools agree, that the closer the marriage of blood relations is, the fewer are the children, the more imperfect in their physical organization, and weaker as to their intellectual capacities. And so imperative were the restrictions imposed on the Jews against marrying their blood kindred, that we must regard it as a means in the Divine Mind of preserving them as a people to the "last times." In no country on earth do we find the Hebrews effeminate in body, or deficient in mental capabilities. On the contrary, they have furnished the world, within the last half century, with the most splendid composers, the most perfect musicians, the most brilliant orators, the most accomplished scholars; they have been found the bravest of the brave on the battle-field, peerless in tragedy, magnificent in song, and in finance without an equal.

What cleanliness, temperance, moderation, and industry did for the Jews in ancient times, they are doing in these last days for a people especially remarkable, a people proverbially "well to do," and as proverbially the longest livers; and who are now patterns to all, as well as the admiration of all, in the neatness of their attire, in the quietude of their lives, and in their universal, substantial thrift (we mean the Quakers); and the world over, there is not to be found a begging "Friend," or a loafing "Israelite."

We have good authority, then, for saying to our readers, that if they would have a pilgrimage, long and healthful, ending in a serene and happy old age, they must obey the Divine precepts of many ages ago: —

"Be ye temperate in all things."

"Wash you, make you clean."

"In the sweat of thy face shalt thou eat bread." For,

"If any would not work, neither should he eat."

Temperance, cleanliness, and industry! This is the Hygiene of the Bible. A "pathy" as old as the race. A system of medication applicable to all climes and all constitutions; always safe, always efficient, and to which human sagacity, in the space of six thousand years, has not added one radically new idea.

